# The effect of transcranial magnetic stimulation on cognitive function in post-stroke patients: a systematic review and meta-analysis

**DOI:** 10.1186/s12883-024-03726-9

**Published:** 2024-07-05

**Authors:** Mingjin Zhu, Siyu Huang, Wenjun Chen, Guoyuan Pan, Yibo Zhou

**Affiliations:** 1 Department of Rehabilitation Medicine, Tongde Hospital of Zhejiang Province, Hangzhou, China; 2 Graduate School, Shanghai Jiaotong University School of Medicine, Shanghai, China; 3 Department of Pharmacy, Xixi Hospital of Hangzhou, Hangzhou, 310023 China

**Keywords:** Stroke, Cognitive impairment, Transcranial magnetic stimulation, Randomized controlled trial, Meta-analysis

## Abstract

**Background and Objective:**

Transcranial magnetic stimulation (TMS) is considered as a promising treatment option for post-stroke cognitive impairment (PSCI).Some meta-analyses have indicated that TMS can be effective in treating cognitive decline in stroke patients, but the quality of the studies included and the methodologies employed were less than satisfactory. Thus, this meta-analysis aimed to evaluate the efficacy and safety of TMS for treating post-stroke cognitive impairment.

**Methods:**

We searched online databases like PubMed, Embase, Cochrane Library, and Web of Science to retrieve randomized controlled trials (RCTs) of TMS for the treatment of patients with PSCI. Two independent reviewers identified relevant literature, extracted purpose-specific data, and the Cochrane Risk of Bias Assessment Scale was utilized to assess the potential for bias in the literature included in this study. Stata 17.0 software was used for data analysis.

**Results:**

A total of 10 studies involving 414 patients were included. The results of the meta-analysis showed that TMS was significantly superior to the control group for improving the overall cognitive function of stroke patients (SMD = 1.17, 95% CI [0.59, 1.75], I^2^ = 86.1%, *P* < 0.001). Subgroup analyses revealed that high-frequency rTMS (HF-rTMS), low-frequency rTMS (LF-rTMS), and intermittent theta burst stimulation (iTBS) all have a beneficial effect on the overall cognitive function of stroke patients. However, another subgroup analysis failed to demonstrate any significant advantage of TMS over the control group in terms of enhancing scores on the Loewenstein Occupational Therapy Cognitive Assessment (LOTCA) and Rivermead Behavioral Memory Test (RBMT) scales. Nonetheless, TMS demonstrated the potential to enhance the recovery of activities of daily living in stroke patients, as indicated by the Modified Barthel Index (MBI) (SMD = 0.76; 95% CI [0.22, 1.30], I^2^ = 52.6%, *P* = 0.121).

**Conclusion:**

This meta-analysis presents evidence supporting the safety and efficacy of TMS as a non-invasive neural modulation tool for improving global cognitive abilities and activities of daily living in stroke patients. However, given the limited number of included studies, further validation of these findings is warranted through large-scale, multi-center, double-blind, high-quality randomized controlled trials.

**PROSPERO registration number:**

CRD42022381034.

**Supplementary Information:**

The online version contains supplementary material available at 10.1186/s12883-024-03726-9.

## Introduction

The prevalence of stroke is increasing due to a growing and aging population, making it is the second most common cause of acquired disability worldwide [1]. Post-stroke cognitive impairment (PSCI) is a frequent complication after stroke, and stroke events significantly increase the risk of developing dementia [2, 3], leading to an earlier onset of dementia by up to 10 years [4]. Cognitive decline is strongly associated with a lower quality of life following a stroke [5] and prolongs hospital stays [6]. The pathogenesis of post-stroke cognitive impairment involves various cellular changes such as disrupted redox state, mitochondrial dysfunction, blood-brain barrier disruption, microglia activation, and amyloid-β deposition in the brain parenchyma [7–10]. Cognitive impairments cannot be solely attributed to the specific locations of stroke but can be caused by damages to anatomically distributed brain networks supporting cognition [11]. Controlling vascular risk factors, drug treatments such as cholinesterase inhibitors and N-methyl-D-aspartate receptor antagonist may improve PSCI [12–14]. These drugs have shown effectiveness in enhancing cognitive functioning but are often accompanied by adverse reactions [15–17]. Currently, there is no approved pharmacological treatment specifically designed for post-stroke cognitive impairment or dementia [18]. Non-pharmacological therapies, such as lifestyle interventions, cognitive training, physical exercise, and acupuncture, are commonly utilized, but their effectiveness is not significant [19–22].

Transcranial magnetic stimulation (TMS), a non-invasive and relatively safe form of brain stimulation, has gained popularity for its ability to selectively induce electric currents in specific cortical regions of the brain through electromagnetic induction [23]. This technique, widely used in neurological and psychiatric rehabilitation, can modulate cortical excitability, either by exciting or inhibiting targeted brain regions [24, 25]. Repetitive transcranial magnetic stimulation (rTMS) and theta burst stimulation (TBS) are two primary types of TMS therapies [26]. High-frequency rTMS (HF-rTMS) is known to increase excitability in the target cortical regions, whereas low-frequency rTMS (LF-rTMS) induces the opposite effect [27]. Intermittent theta burst stimulation (iTBS) delivers short bursts of high-frequency pulses intermittently to enhance cortical excitability, while continuous theta burst stimulation (cTBS) applies continuous pulses at a lower frequency to inhibit cortical activity [28]. Both iTBS and cTBS are types of rTMS utilized for neuro-modulation in clinical settings.

rTMS has undergone extensive research in patients with Alzheimer’s disease (AD) and has emerged as an effective treatment for cognitive impairment associated with AD, offering safe and long-lasting effects [29, 30]. Studies have shown that rTMS can mitigate cognitive deficits in AD mice by inhibiting apoptosis through the activation of the cAMP/PKA/CREB signaling pathway [31]. The application of iTBS has demonstrated beneficial effects on depression, executive function, and target engagement of the cognitive control network in older adults [32]. iTBS, acknowledged as a time-saving and cost-effective repetitive transcranial magnetic stimulation regime, has shown promise in animal experiments for improving cognitive decline and alleviating AD-type pathology in APP/PS1 mice [33]. iTBS is regarded as a modified design of rTMS that can serve as a complementary approach to psychotherapy [34].

Previous studies have demonstrated the effectiveness of TMS in patients with post-stroke cognitive impairment [35, 36]. Some researchers have also reported that high-frequency rTMS may not have a discernible impact on cognition in post-stroke patients [37]. Recently, a systematic review and meta-analysis indicated that rTMS is an effective technique for treating post-stroke patients with cognitive impairment [38]. However, it is essential to acknowledge that certain studies included in the meta-analysis were published quite some time ago, potentially compromising the quality of the literature. Furthermore, the meta-analysis relied on comparing final values, which is less efficient and robust than utilizing change scores between baseline and post-intervention measurements [39]. Therefore, our aim is to conduct an updated meta-analysis to assess the effects of TMS on cognitive function in post-stroke patients.

## Materials and methods

This meta-analysis was conducted according to the guidelines of the Preferred Reporting Items for Systematic Reviews and Meta-Analyses (PRISMA) [40]. This study was prospectively registered with the PROSPERO database of systematic reviews (CRD42022381034): https://www.crd.york.ac.uk/prospero/display_record.php?ID=CRD42022381034.

### Literature search strategy

We searched four databases (Pubmed, Embase, Cochrane Library, and Web of Science) for published randomized controlled trials (RCTs) from database inception up to May 2024. The search strategy used the following terms: ((((((cerebral OR cerebellar OR intracerebral OR intracranial OR brain OR cerebrovascular) AND (bleed* OR haemorrhage* OR hemorrhage* OR infarction* OR occlusion* OR emboli* OR embolus OR thrombus OR thrombosis OR thrombi)) OR (stroke OR apoplexy OR post stroke OR post-stroke))) AND (cognitive OR cognition OR dementia OR processing OR attention OR language OR visuospatial OR memory OR executive function OR intelligence)) AND (transcranial magnetic stimulation OR TMS OR rTMS OR theta burst stimulation OR TBS OR iTBS OR cTBS)) AND (randomized controlled trial)).

### Inclusion and exclusion criteria

We formulated the literature inclusion criteria by the principles of PICOS (Population, Intervention, Comparison, Outcomes and Study): (1) participants diagnosed with stroke, (2) cognitive impairment at least one domain in attention and executive, memory, language and visuospatial function caused by stroke, (3) including intervention group (rTMS or iTBS) and control group (Sham or no stimulation), (4) outcomes including cognitive function assessment, and complete clinical data was provided in the literature, (5) randomized controlled trial, (6) participants were adults (≥ 18 years), (7) articles published in English language, (8) studies in recent 10 years. The exclusion criteria were: (1) severe cognitive decline that impedes cooperation, (2) cognitive impairment or dementia before stroke, (3) data sets that were incomplete and unable to be analyzed, even after attempting to contact the authors via email, and in cases where there were articles from the same study, previous and incomplete data will be excluded.

### Data extraction

Two reviewers (S.H. and W.C.) working independently examined and extracted data from each included study. The extracted information included (1) general characteristics: author, year of publication, study design, number of participants, mean age, stroke duration; (2) intervention: type of stimulation, location of stimulation, intensity, frequency, total pulses per treatment, sham stimulation method, intervention time and adverse effects; (3) statistical data of the score of cognitive performance. Any discrepancies in data obtained by the two reviewers were resolved by discussing with another professional researcher to reach a consensus.

### Quality of studies and risk of bias assessment

The quality of included studies was assessed using the Cochrane Handbook for Systematic Reviews of Interventions [41]. The following characteristics were assessed: (1) random sequence generation; (2) allocation concealment; (3) blinding; (4) handling of incomplete outcome data; (5) evidence of selective outcome reporting; (6) other potential risks that could impact the validity of the study. The risk of bias for each criterion was categorized as low, high, or unclear.

### Statistical analysis

Stata 17.0 software was used for Meta-analysis. Cochrane Rev-Man 5.4 software was used for quality assessment. Homogeneity test (Q test) and I^2^ value was used to test the heterogeneity of the included research. The effect of TMS on cognitive function in post-stroke patients was defined as the mean difference (MD) in the change of cognitive indicators relative to baseline (before stimulus treatment) in the experimental and control groups. Given the diversity of cognitive indicators applied in the included studies, standardized mean difference (SMD) and 95% confidence intervals were used to summarize eligible trial pooled effect sizes. SMD is often used in meta-analysis to compare mean differences between groups with outcome variables measured on different scales. Because two studies [42, 43] did not show a net change of cognitive scores between baseline and post intervention, the following formulas were used:

Mean changes = Mean post − Mean baseline;

$$\begin{gathered}{\text{SD}}\,{\text{change}} = \sqrt {{\text{SD}}_{{\text{baseline}}}^2 + {\text{SD}}_{{\text{post}}}^2 - (2 \times {\text{coefficent}} \times {\text{SD baseline}} \times {\text{SD post}})} \\ \end{gathered}$$

If this correlation coefficient is unknown, it may be estimated as 0.5. If there is a similar study that reports summary statistics for change from baseline, baseline and final values, a better estimate (Chap. 6.5.2.8, Cochrane Handbook) of the correlation coefficient is:

$$\text{coefficent}=\frac{{\text{SD}}_{\text{baseline}}^{2}+{\text{SD}}_{\text{post}}^{2}-{\text{SD}}_{\text{Change}}^{2}}{2\times {\text{SD}}_{\text{baseline}}{\text{SD}}_{\text{post}}}$$

In some studies, the standard errors of the mean or standard deviations were not given but figures, which had to be recalculated. In the case of one included study [44], the approximate data were extracted from figures in this paper using the online version of the web-based WebPlotDigitizer (https://apps.automeris.io/wpd/index.zh_CN.html, Copyright 2010–2022 Ankit Rohatgi) software.

Heterogeneity was quantified using the I^2^ statistic, and I^2^ ≤ 50% was considered low heterogeneity, then the meta-analysis was conducted with fixed effects model. I^2^ > 50% indicated substantial heterogeneity, and the random effects model was adopted for meta-analysis. In addition, high statistical heterogeneity was analyzed by subgroup analysis.

Sensitivity analysis was also used to explore the source of heterogeneity, and funnel plot, Begg’s and Egger’s tests were performed to evaluate publication bias. Statistical significance was considered for p-values less than 0.05.

## Result

### Search results

**Fig. 1:**
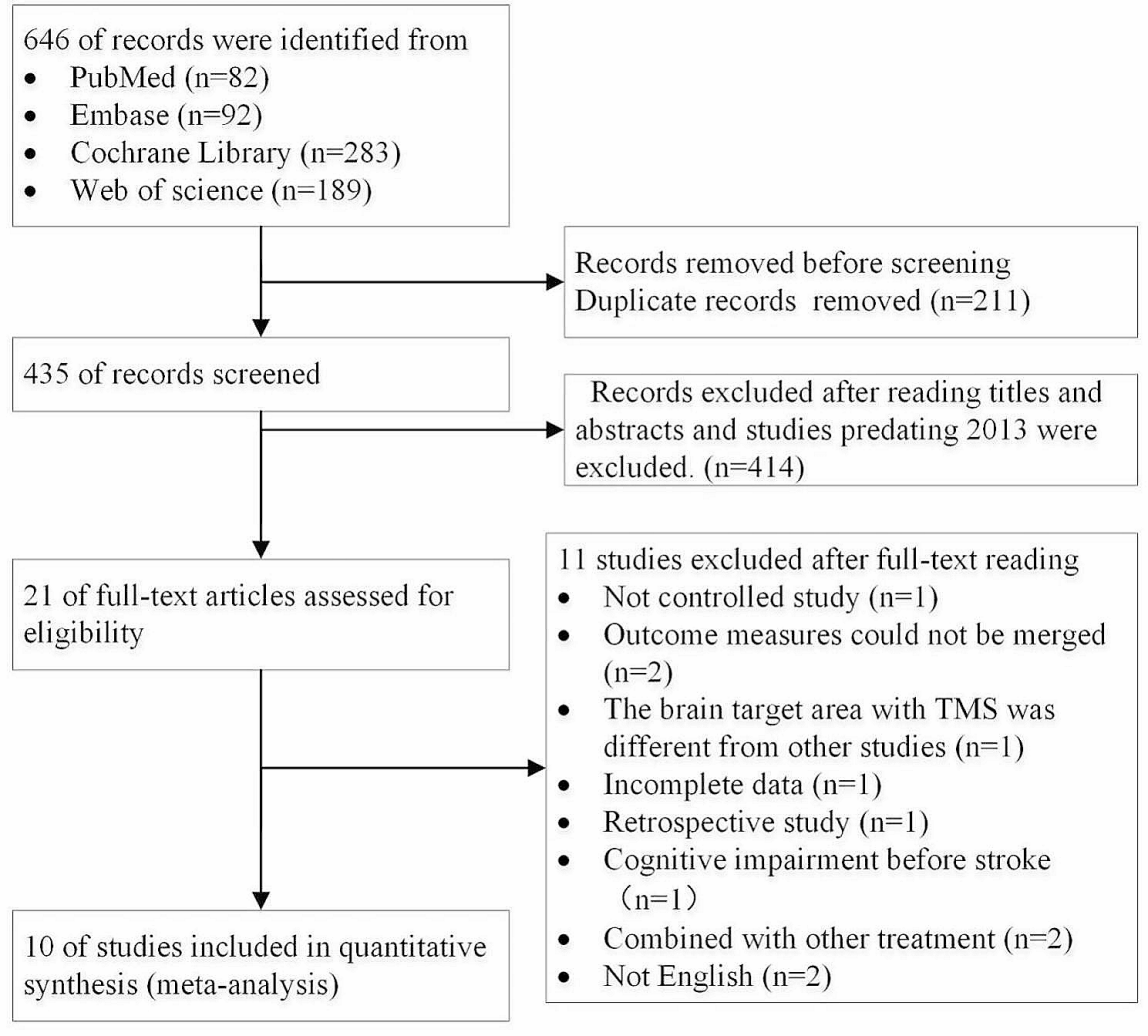
Flow chart for study screening

The initial search identified a total of 646 records, and 435 studies remained after excluding 211 duplicate records. Of these, 435 studies were excluded after reading titles and abstracts (including studies published more than 10 years ago). Two reviewers (S.H. and W.C.) independently read the full-text articles of the 21 studies, and 11 studies were excluded. Eventually, 10 randomized controlled trials were included in this meta-analysis [43–52]. Figure 1 shows a flowchart of screening and selection process.

### Study characteristics

Ten studies were included in this meta-analysis, comprising a total of 414 participants. The characteristics of the included studies are presented in Table 1.All patients included in this review had a diagnosis of stroke, and their cognitive function was assessed. The experimental group in seven studies received rTMS treatment, and three studies received iTBS. One trial included 2 intervention arms and one control group (rTMS vs. iTBS vs. control) [50]. The TMS stimulation location in eight studies [43–45, 47–50, 52] was the left dorsal lateral prefrontal cortex (DLPFC) with high-frequency rTMS (HF-rTMS) stimulation (≥ 5 Hz) or iTBS, and the remaining two studies were right DLPFC and contralateral DLPFC with low-frequency rTMS (LF-rTMS) stimulation (1 Hz). The details of each study are provided in Table 1.

**Table 1:** Basic characteristics of the included studies

Study-year	Study design	Random sequence/ concealment	*n*	Mean Age	Mean time post stroke (month)	Stroke type (ischemic, hemorrage)	Side of lesion (right, left, bilateral)	Handess	Site of stimulation	Type of stimulation	Time (Day)	Outcome
Park et al. 2015	RCT, blind NC C: no stimulation	NC	10/10	NC	NC	(8,2)/(4,6)	NC	right-handed	left DLPFC	rTMS, 10 Hz, 1000 pulses	12	MMSE, LOTCA
Lu et al. 2015	RCT, single-blind C: sham stimulation	random number table	19/21	42.5/47.3	2.23/1.87	(8,11)/(10,11)	(8,11,0)/(10,11,0)	NC	right DLPFC	rTMS, 1 Hz, 600 pulses	20	MoCA, LOTCA, RBMT
Yin et al. 2020	RCT, blind NA C: no stimulation	computer-generated	16/18	56.69/58.17	1.73/1.83	(11,5)/(12,6)	(6,4,6)/(7,6,5)	right-handed	left DLPFC	rTMS, 10 Hz, 700 pulses	10, 20	MoCA, RBMT, MBI
Li, Luo et al. 2020	RCT, double-blind C: sham stimulation	random number table	15/15	65.47/65.43	0.76/0.64	(0,15)/(0,15)	(5,10,0)/(6,9,0)	right-handed	left DLPFC	rTMS, 5 Hz, 2000 pulses	15	MoCA, MMSE
Liu et al. 2020	RCT, double-blind C: sham stimulation	random number table	29/29	58.55/57.69	8.79/8.62	(20,9)/(15,14)	(18,11,0)/(15,14,0)	NC	left DLPFC	rTMS, 10 Hz, 700 pulses	20	MMSE
Tsai et al. 2020	RCT, double-blind C: sham stimulation	computer-generated, sealed evelopes	15/15	60.13/56.23	18.47/38	(7,8)/(10,5)	all located in the left	NC	left DLPFC	iTBS, 50 Hz, 600 pulses	10	RBANS
			11/15	57.45/56.23	33.27/38	(3,8)/(10,5)			left DLPFC	rTMS, 5 Hz, 600 pulses	10	
Li, Ma et al. 2021	RCT, single-blind C: sham stimulation	random number table	33/22	61.79/59.47	0.95/0.93	NC	NC	right-handed	contralateral DLPFC	rTMS, 1 Hz, 1000 pulses	5	MoCA, MBI
Li, Wen et al. 2022	RCT, double-blind C: sham stimulation	randomized table, sealed envelopes	28/30	69.5/66.0	0.83/0.83	(18,10)/(14,16)	(12,16,0)/(6,24,0)	NC	left DLPFC	iTBS, 50 Hz, 600 pulses	10	MMSE
Chu et al. 2022	RCT, single-blind C: no stimulation	randomized table	21/20	57.24/66.75	4/6	(13,8)/(12,8)	(9,12,0)/(7,12,0)	NC	left DLPFC	iTBS, 50 Hz, 600 pulses	30	MMSE, LOTCA, MBI
Zhang et al. 2024	RCT, single-blind, C: no stimulation	NC	19/18	58.00/69.94	4/6	NC	NC	NC	left DLPFC	iTBS, 50 Hz, 600 pulses	30	LOTCA, MBI

RCT: randomized controlled trial; NC: not clear; DLPFC: dorsal lateral prefrontal cortex; MMSE: mini-mental state examination; MoCA: Montreal cognitive assessment; LOTCA: Loewenstein occupational therapy cognitive assessment; RBMT: Rivermead behavioral memory test; MBI: modified Barthel index; RBANS: repeatable battery for the assessment of neuropsychological Status

### Study quality

**Fig. 2:**
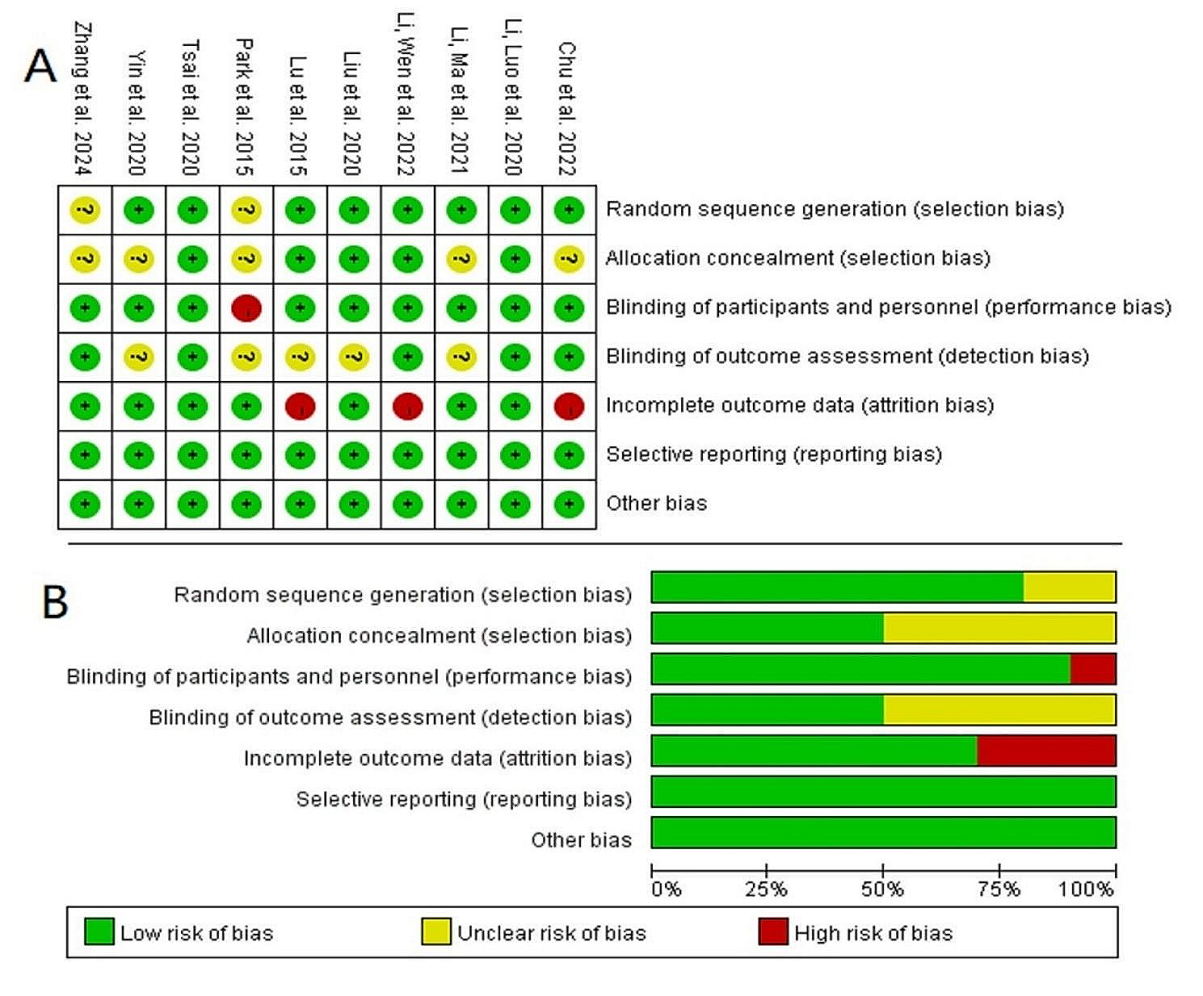
Quality assessment of selected studies by the Cochrane risk of bias tool

**Fig. 3:**
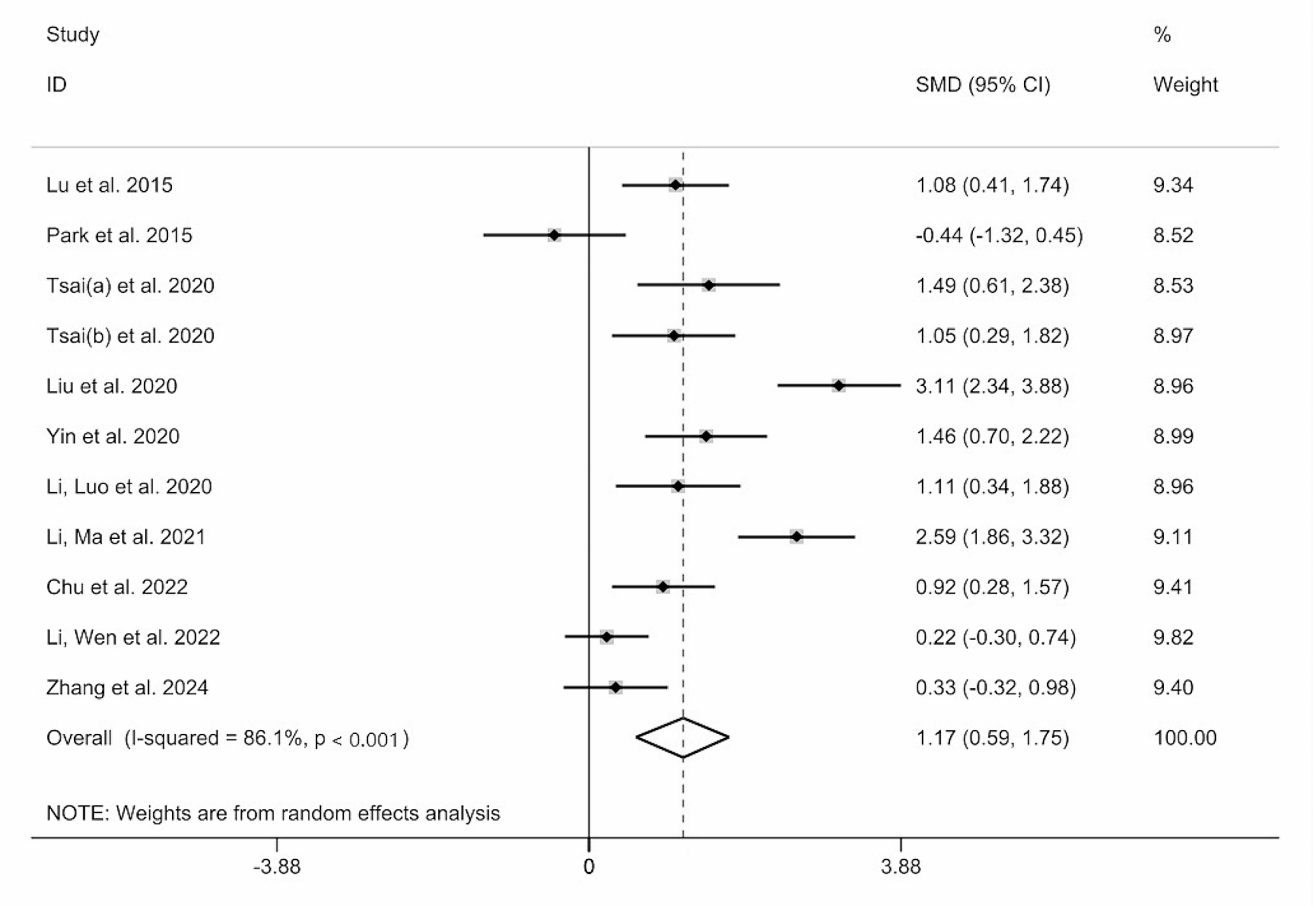
A forest plot of the effect of TMS on cognitive function in stroke patients. Abbreviations: SMD Standardized mean differences, CI confidence intervals

Risk of bias in the included studies was evaluated using Cochrane’s risk of bias tool [53]. The results were as illustrated in Fig. 2.All studies in this review were RCTs. Four studies [48–50, 52] were double blinded and the other four were single blinded [43, 44, 46, 49]. Two studies did not mention if blinded [45, 47]. The control group in 6 studies included sham stimulation [46, 48–52]. Eight studies described random sequences generated using random number tables or computer programs [44, 46–52]. Two studies reported allocation procedures with concealment [50, 52]. Therefore, all included studies were considered to have a mild risk of bias (Fig. 3).

(**A**) Risk of bias summary: review authors’ judgments about each risk of bias item for each included study. (**B**) Risk of bias graph: review authors’ judgements about each risk of bias item presented as percentages across all included studies.

### Effect of TMS on cognition in stroke patients

Global cognition was measured by the Mini-Mental Status Examination (MMSE), Montreal Cognitive Assessment (MoCA) scale in this study, Loewenstein Occupational Therapy Cognitive Assessment (LOTCA), Repeatable Battery for the Assessment of Neuropsychological Status (RBANS). MMSE and MoCA are both valid cognitive tools in stroke patients [54]. LOTCA is a relatively systematic assessment method in evaluating cognitive function in patients with stroke, and is slightly better than the MMSE [55]. The RBANS is a widely used brief test for detecting cognitive impairment in various neuropsychiatric conditions, which has also been applied to assess cognitive function in stroke patients [56].

A total of 10 RCTs (N_TMS_=216, N_control_=213) were included in the pooled meta-analysis to access the effects of TMS vs. sham/no stimulation on global cognition in stroke patients. The improvement in global cognition among stroke patients was significantly greater in TMS group compared to the control group (SMD = 1.17, 95% CI [0.59, 1.75], I^2^ = 86.1%, *P* < 0.001) (Fig. 3), and random effects model was used because of substantial heterogeneity.

### Subgroup analysis of the effect of HF-rTMS, LF-rTMS and iTBS

**Fig. 4:**
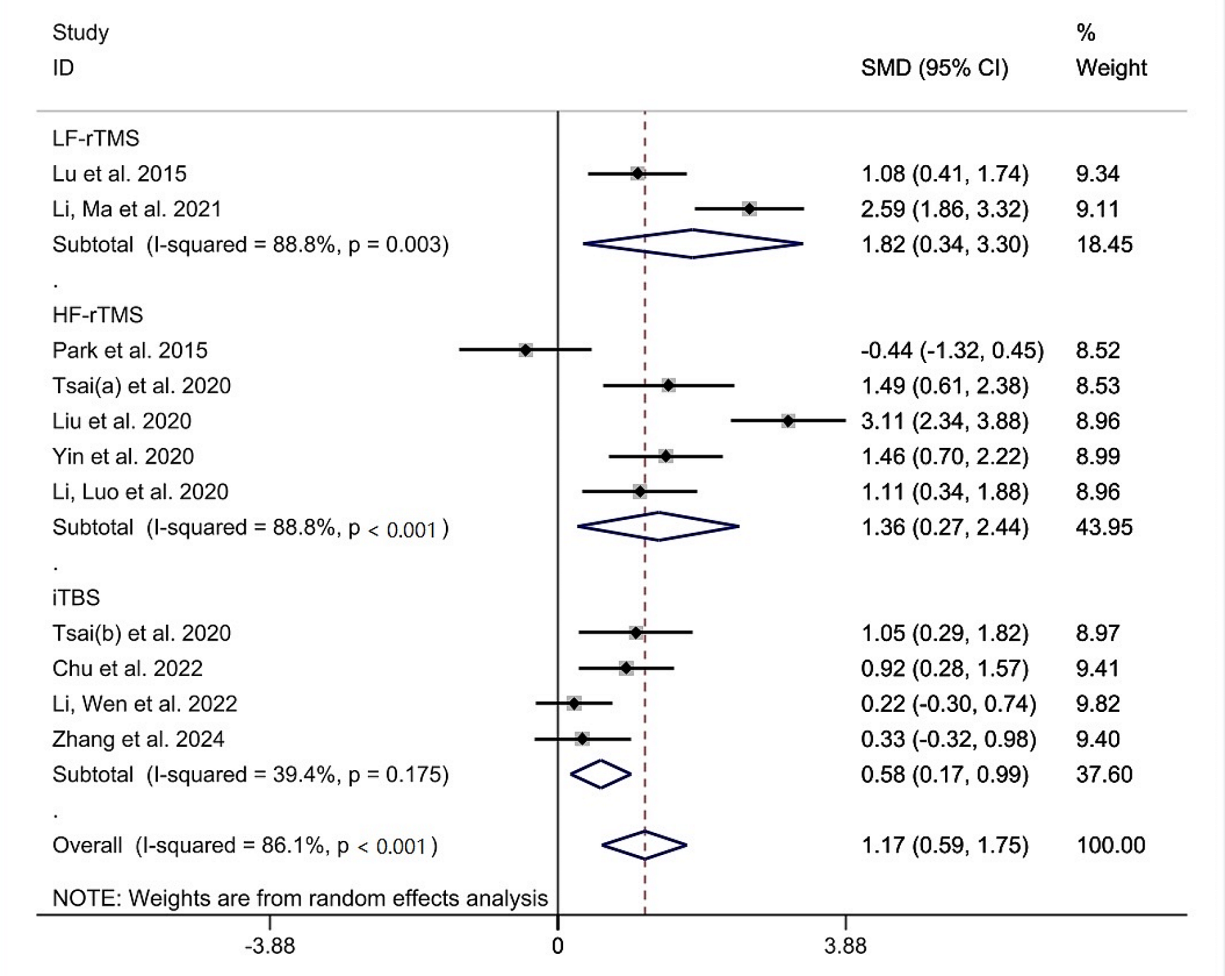
Subgroup analysis of LF-rTMS, HF-rTMS and iTBS on cognitive function in stroke patients. Abbreviations: SMD Standardized mean differences, CI confidence intervals, LF-rTMS low-frequency repetitive transcranial magnetic stimulation, HF-rTMS High-frequency repetitive transcranial magnetic stimulation, iTBS intermittent theta burst stimulation

Subgroup analyses were performed based on stimulation types (HF-rTMS, LF-rTMS, and iTBS). The results revealed that LF-rTMS had an SMD of 1.82(95%CI [0.34, 3.30], I^2^ = 88.8%, *P* = 0.003) and HF-rTMS had an SMD of 1.36(95%CI [0.27, 2.44], I^2^ = 88.8%, *P* < 0.001). The SMD between trials in iTBS group was 0.58 (95%CI [0.17, 0.99], I^2^ = 39.4%, *P* = 0.175). Subgroup analyses revealed that all forms of TMS yielded a positive effect on the global cognitive function of stroke patients (Fig. 4).

### Subgroup analysis of the effect of TMS on LOTCA, RBMT, MBI

**Fig. 5:**
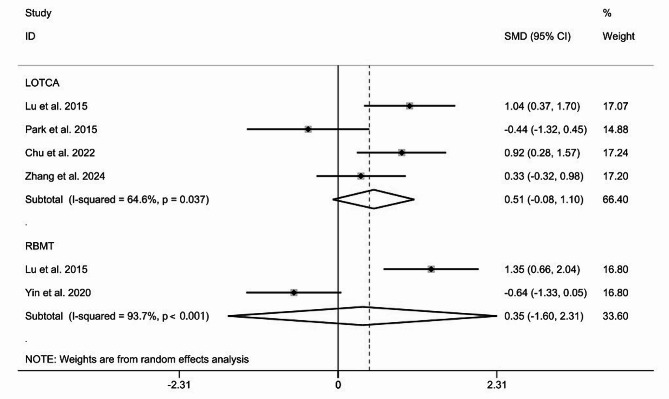
Subgroup analysis of TMS on the improvement of LOTCA, RBMT scores. Abbreviations: LOTCA Loewenstein occupational therapy cognitive assessment, RBMT Rivermead behavioral memory test, SMD standardized mean differences, CI confidence intervals

**Fig. 6:**
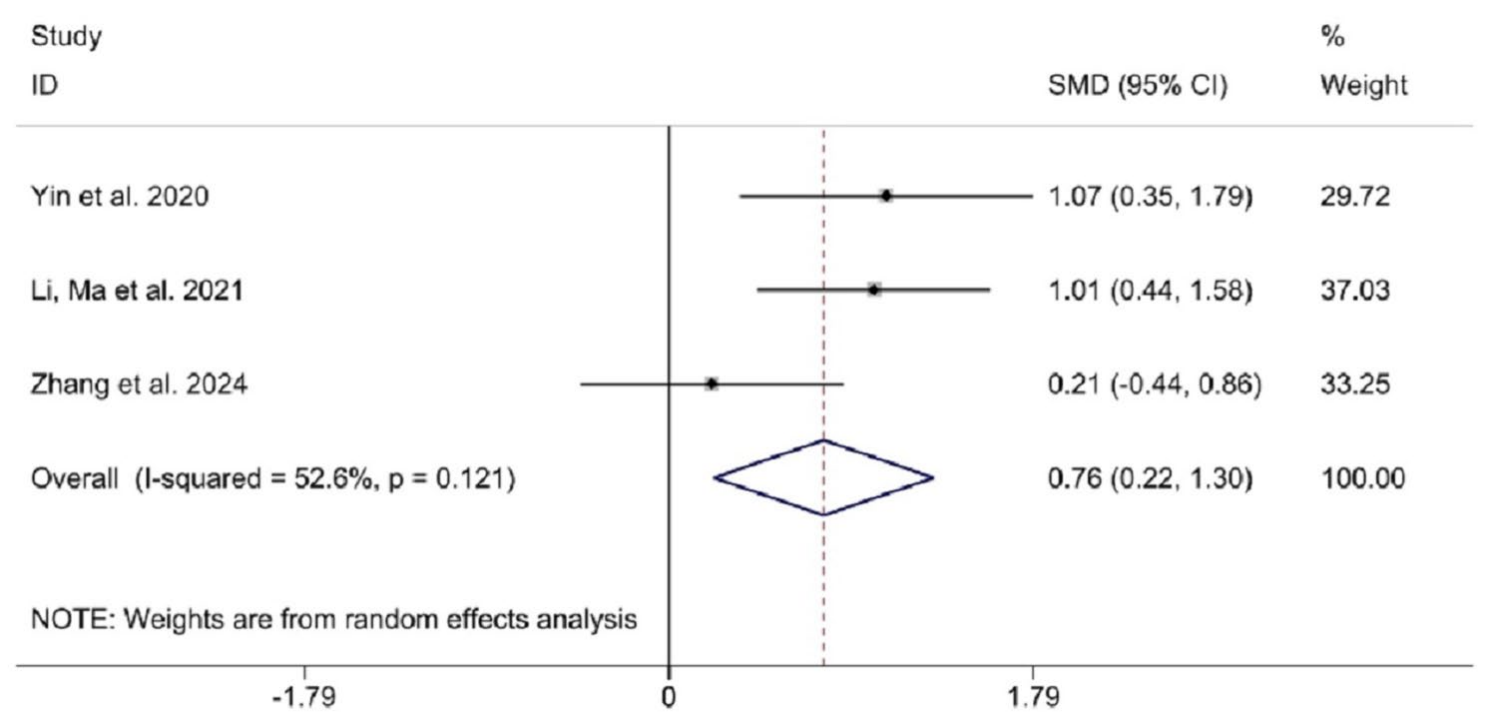
Subgroup analysis of TMS on the improvement of MBI. Abbreviations: MBI modified Barthel index, SMD standardized mean differences, CI confidence intervals

We also performed subgroup analysis of selected outcomes (LOTCA, RBMT, and MBI) and compared the influence of TMS treatment on the recovery of executive capacity, memory, and activity of daily living in patients with stroke. LOTCA is a series of tests designed for occupational therapists, to assess a person’s cognitive processing ability and to determine whether a person is able to carry out everyday functional tasks [57]. The Rivermead Behavioural Memory Test (RBMT) was designed specifically to evaluate memory abilities for the performance of daily tasks [58]. The Modified Barthel Index (MBI) is a commonly used scale that measure disability or dependence in activities of daily living in stroke patients [59].Three studies reported LOTCA scores and two studies reported RBMT. The pooled results did not reveal that TMS was better than control group on the improvement of LOTCA and RBMT scores (SMD = 0.51; 95% CI [-0.08, 1.10], I^2^ = 64.6%, *P* = 0.037, and SMD = 0.35, 95% CI [-1.60, 2.31], I^2^ = 93.7%, *P* < 0.001) with random-effect model (Fig. 5). However, when it came to the changes of MBI scores, the difference between the two groups was statistically significant according to the analyses (SMD = 0.76; 95% CI [0.22, 1.30], I^2^ = 52.6%, *P* = 0.121), which indicated that TMS improved the MBI scores more efficiently (Fig. 6).

### Publication bias and sensitivity analysis

**Fig. 7:**
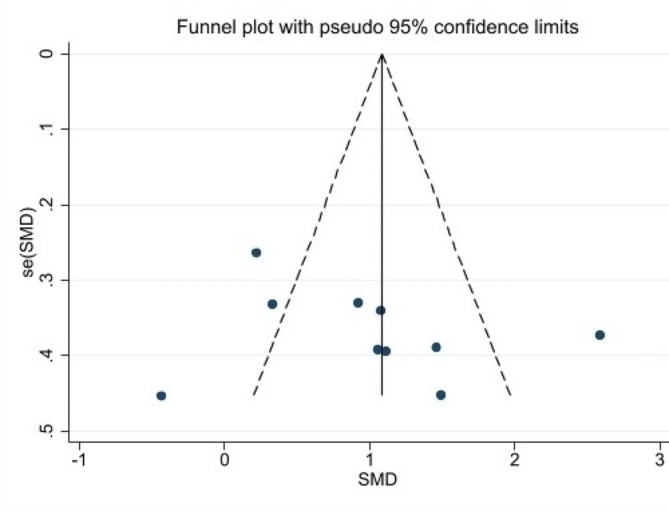
Begg’s funnel plot

**Fig. 8:**
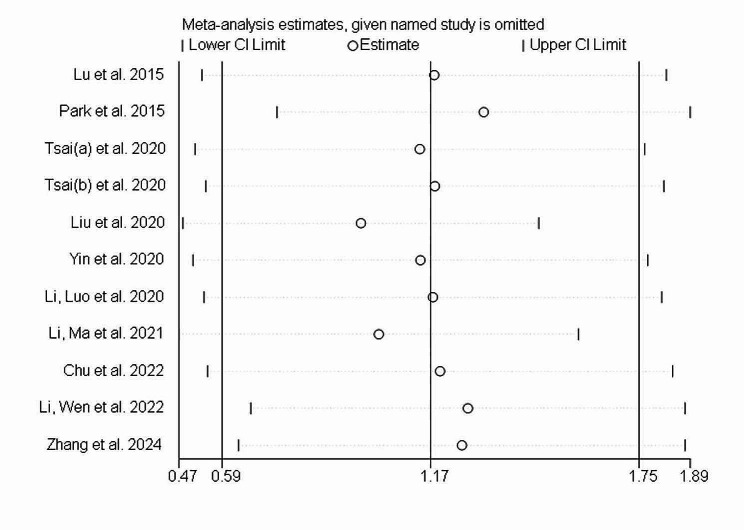
Sensitivity analysis

Egger’s regression tests were conducted to evaluate the influence of small studies as publication bias. The P-value of Egger’s test do not support the existence of publication bias for all interventions (t = 1.18, *P* = 0.269). The shape of Begg’s funnel plot seems to be asymmetric (Pr >|z|=0.213) (Fig. 7). To identify the influence of individual studies on the overall meta-analysis, a sensitivity analysis was conducted. This involved systematically omitting each study to evaluate its impact on the collective results. The analysis revealed that no single study significantly affected the overall effect sizes (Fig. 8). Thus, our meta-analysis was relatively stable.

### Adverse reaction

TMS is a noninvasive form of brain stimulation. Generally, TMS is considered safe and well-tolerated. In this systematic review, only three included studies reported adverse effects [43, 45, 46, 48]. Those patients in TMS groups felt discomfort because of the unique sounds that occur during stimulation and facial muscle contractions [45]. Three studies showed that several patients experienced transient headaches or dizziness in the TMS group, and patients in sham or control group complained light dizziness or headache [43, 46, 48]. However, these symptoms disappeared quickly without any specific interventions. These symptoms disappeared quickly without any specific interventions.

## Discussion

This comprehensive meta-analysis involved an in-depth review of ten randomized controlled trials, showing the significant superiority of TMS over the control group in enhancing the overall cognitive function of stroke patients, while presenting minimal adverse reactions. These findings not only align with but also reinforce previous meta-analytic results in this field. Moreover, TMS demonstrated potential in improving the recovery of activities of daily living in stroke patients. However, subgroup analysis did not reveal a clear advantage of TMS over the control group in terms of enhancing scores on the LOTCA and RBMT scales, indicating the necessity for further research in this area.

Many stroke patients in the process of recovery often encounter various cognitive deficits, such as difficulties in attention, memory, executive functioning, and information processing [60]. The occurrence of cognitive impairment is closely related to the damage of specific brain regions such as the frontal lobe, anterior temporal lobe, cingulate gyrus, and hippocampus [61]. Cerebral ischemic injury-induced cognitive impairment involves numerous signaling pathways. Various transcription factors, intracellular adhesion molecules, and endogenous growth factors play a role in the pathogenesis of stroke-related cognitive impairment, offering potential therapeutic targets for treatment [62]. Preclinical mechanisms for cognitive function improvement after stroke include neuroplasticity, angiogenesis, inflammatory response modulation, and neurotrophic factor activity. These processes contribute to brain repair, synaptic rewiring, and functional recovery [63, 64]. TMS is a non-invasive technique that targets specific areas of the cerebral cortex. TMS studies have provided valuable insights into the pathophysiology of neurodegenerative disorders and stroke, further enhancing our understanding of post-stroke brain reorganization [65].

The results of our study indicated a significant improvement in global cognitive function with the use of TMS in stroke patients. The global cognitive assessment tools used in this study included MMSE, MoCA, and RBANS. Although MMSE and MOCA are two cognitive screening tools, many studies use these two scales to assess patient’s cognitive status before and after treatment for clinical trial or practice [66]. In this study, the intervention types of TMS included HF-rTMS, LF-rTMS, and iTBS. Subgroup analyses revealed that all three TMS treatments had a positive impact on global cognitive function in stroke patients. According to the theory of imbalanced interhemispheric interactions induced by stroke [67], HF-rTMS and iTBS protocols were considered “excitatory”, while LF-rTMS was considered ‘‘inhibitory” [68]. The dorsolateral prefrontal cortex (DLPFC) plays a critical role in cognitive control, and applying TMS to the DLPFC can enhance cognitive processing [69]. The left DLPFC has been linked to the regulation of stress-related cognitive processes and physiological responses [61, 70]. Among the selected studies, eight chose the left DLPFC as the stimulation site, while the remaining studies focused on the right or contralateral DLPFC. The excitatory stimulation of the left DLPFC and inhibitory stimulation of the right DLPFC may enhance cognitive function in stroke patients, with potential dependence on handedness. Research confirms that the dominant DLPFC hemisphere is typically located in the left hemisphere for the majority of right-handed individuals, while 16.7% of left-handed individuals also exhibit left-sided dominance in their DLPFC hemisphere [71]. Subgroup analysis indicated that there was no statistically significant difference between the two groups when using the LOTCA and RBMT scales. The LOTCA test involves multiple cognitive tasks and typically takes around 45 minutes to complete. LOTCA is considered to be a time-consuming and demanding tool, offering a more comprehensive assessment compared to other cognitive evaluations like the MMSE or MoCA [72]. In the subgroup analysis, it was not conclusively demonstrated that TMS intervention led to a superior improvement in patients’ LOTCA scores, thus further research is needed to confirm this. The RBMT, on the other hand, is specifically designed to detect impairment in everyday memory function, which includes various domains of memory function such as immediate memory, delayed memory, recognition memory, prospective memory, visual memory, verbal memory, spatial memory, and orientation [73]. Some researchers have found that TMS has a limited effect on working memory in patients with brain disorders [74]. Another study did not observe a significant effect of TMS on working memory in patients with Alzheimer’s disease [75]. In our study, we observed that the effect of TMS on memory improvement was not superior to that of the control group. TMS may primarily improve cognitive function in stroke patients by enhancing their executive function [52, 76]. Research has shown that TMS can enhance mental flexibility and task-switching abilities in the executive function of patients with mild cognitive impairment [77]. In terms of the daily living abilities of stroke patients, our study found that TMS can significantly improve their Barthel Index scores compared to the control group, indicating that TMS can enhance their ability to perform daily task. Although the MBI does not directly measure cognitive function, it does reflect a patient’s level of independence in performing these tasks, which can be influenced by cognitive impairment. TMS may potentially reduce the risk of depression in post-stroke patients, thereby further enhancing their daily life capabilities [78]. When it comes to adverse reactions, TMS therapy generally demonstrates good safety. The most common side effects were headache, fatigue, and pain/discomfort at the stimulation site [79], which are typically mild and easily manageable. A rare but serious adverse event of TMS treatment is seizure [80]. In this study, we observed that adverse reactions to TMS involved headache and dizziness, both of which promptly resolved without the need for specific interventions.

### Limitation

Our meta-analysis applied strict inclusion and exclusion criteria. However, this study does have several limitations: (a) Variations in stimulation frequency and intervention duration existed among the included studies, and most of the studies had relatively small sample sizes. (b) The randomized controlled trials lacked standardization. Some studies did not include sham stimulation as negative controls, and there were instances where allocation concealment or blinding was not properly implemented. (c) Differences in participant age and variations in the severity of their illnesses may have influenced the rehabilitation outcomes. (d) The effectiveness of TMS administration in these studies may not be definitively confirmed due to the limited number of available studies. Given these limitations, it is important to note that the conclusions drawn from this meta-analysis may be affected.

## Conclusion

Overall, this meta-analysis has shown that TMS is a safe and effective non-invasive neural modulation tool in the treatment of post-stroke cognitive impairment. TMS has shown significant improvements not only in global cognitive abilities but also in activities of daily living for stroke patients. However, it is worth noting that TMS has been linked to certain adverse effects, such as headaches or dizziness. Further research involving larger sample sizes and improved experimental design is still required to determine the optimal therapeutic protocol and validate the benefits of TMS in treating post-stroke cognitive impairment.

### Electronic supplementary material

Below is the link to the electronic supplementary material.

Supplementary Material 1

Supplementary Material 2

## Data Availability

All the data analyzed during this study are included in this article. Further inquiries can be directed to the corresponding author.

## Abbreviations

TMS: Transcranial magnetic stimulation
rTMS: Repetitive transcranial magnetic stimulation
iTBS: Intermittent theta burst stimulation
cTBS: Continuous theta burst stimulation
RCT: Randomized controlled trial
PSCI: Post-stroke cognitive impairment
AD: Alzheimer’s disease
MMSE: Mini-mental state examination
MoCA: Montreal cognitive assessment
LOTCA: Loewenstein occupational therapy cognitive assessment
MBI: Modified Barthel index
SMD: Standardized mean differences
CI: Confidence intervals
RBANS: Repeatable battery for the assessment of neuropsychological status
RBMT: Rivermead behavioral memory test
DLPFC: Dorsolateral prefrontal cortex
PRISMA: Preferred reporting items for systematic reviews and meta-analyses
NC: Not clear
